# Linear regression prediction model for an outcome of Alzheimer's Disease in Down Syndrome including BPSD‐DSII sections and Alzheimer's Disease fluid biomarkers

**DOI:** 10.1002/alz70856_104824

**Published:** 2026-01-07

**Authors:** Charlotte Jacob, Marleen Tollenaere, Erik Fransen, Peter Paul De Deyn, Debby Van Dam, Laboratory of Neurochemistry and Behavior

**Affiliations:** ^1^ University of Antwerpen, Antwerpen, Antwerpen, Belgium; ^2^ Department of Neurology and Memory Clinic, Hospital Network Antwerp (ZNA) Middelheim and Hoge Beuken, Antwerp, Belgium; ^3^ University of GrÖningen, University Medical Center Groningen (UMCG), Department of Neurology and Alzheimer Research Center, GrÖningen, Netherlands

## Abstract

**Background:**

By age 40, virtually all individuals with Down Syndrome (DS) will have developed the pathological symptoms of Alzheimer's Disease (AD) (McCarron, McCallion et al. 2017), making it the genetic leading cause of AD worldwide. Our lab developed a specialized behavior‐based scale to aid in the diagnosis of AD in DS, the BPSD‐DSII (Dekker, Ulgiati et al. 2021). Individuals with DS with or without (questionable) AD (total = 157) were tested with the BPSD‐DSII, and subsequently, we also performed immune‐based assays (Simoa) to further characterize blood biomarkers in this cohort. Three years after the original BPSD‐DSII was administered in this cohort, the individuals were, again, categorized in percentiles based on the BPSD‐DSII behavior frequency changes. The outcome of the different sections of the BPSD‐DSII at baseline and the levels of several blood‐based biomarkers at baseline were put in a statistical prediction model based on linear regression.

**Method:**

Linear regression prediction model coded in R with R studio

**Result:**

The outcome of several sections of the BPSD‐DSII questionnaire, as well as pTau181 levels and GFAP levels, were predictive of percentiles indicating AD in DS.

**Conclusion:**

Specific behavioral changes at baseline such as in apathetic behavior, depressive behavior, and eating and drinking behavior, as well as pTau181 and GFAP levels were predictive for the development of AD in DS.

